# Grafting-Induced Structural Ordering of Lactide Chains

**DOI:** 10.3390/polym11122056

**Published:** 2019-12-11

**Authors:** Artyom D. Glova, Sofya D. Melnikova, Anna A. Mercurieva, Sergey V. Larin, Sergey V. Lyulin

**Affiliations:** 1 Institute of Macromolecular Compounds, Russian Academy of Sciences, Bolshoj pr. 31 (V.O.), 199004 St. Petersburg, Russia; glova@imc.macro.ru (A.D.G.); anna@macro.ru (A.A.M.); selarin@macro.ru (S.V.L.); 2 Institute of Physics, Nanotechnology and Telecommunications, Peter the Great St. Petersburg Polytechnic University, Polytechnicheskaya st. 29, 195251 St. Petersburg, Russia; sofya_m1@mail.ru

**Keywords:** lactide, dipolar chains, polymer brushes, nanocomposites, structural ordering, molecular dynamics, simulations

## Abstract

The structure of a grafted layer of lactide chains in the “dry brush” regime immersed in a melt of chemically similar polymer was examined while varying graft lengths. To this end, microsecond atomistic molecular dynamics simulations were performed. Almost no influence of graft length on the fraction of the grafted chains backfolded to the grafting surface was found. However, a structural ordering was unexpectedly observed in the system when the length of the grafted lactide chains was close to approximately 10 Kuhn segments. This ordering of the grafts is characterized by the formation of helical fragments whose structure is in good agreement with the experimental data for the *α* crystal of the lactide chains. Both the backfolding and the structural ordering may be viewed as the initial stage of the crystallization of the layer of grafted lactide chains. In contrast to the known behavior for conventional polymer brushes in the “dry brush” regime, the structure of the grafted lactide chains can be either amorphous or ordered, depending on the graft length *N* and the grafting density *σ* when their product *Nσ* is fixed.

## 1. Introduction

Due to their importance for a broad variety of applications, including those of automotive, biomedical and packaging, polymer nanocomposites have become a practically and fundamentally attractive class of materials in polymer physics and chemistry. A critical bottleneck in extending the use of these materials is the control of the spatial distribution of nanofillers [1,2]. To obtain the desired property enhancements for the final application, the possible aggregation of nanofillers that is mediated by their poor compatibility with polymer binders must be addressed [1,3]. Grafting polymers or oligomers onto nanofillers, i.e., forming brushes, can significantly enrich the aggregation behavior of the modified nanofillers [4,5,6,7]. The modification of the brush structure has emerged as a common strategy to tailor both the compatibility and the spatial distribution of nanofillers within nanocomposites [4,5,8]. A primary issue, however, is understanding the brush structure in the nanocomposites that are filled with chain-grafted nanofillers, which remains an active and challenging research area [7,8,9,10,11,12,13,14].

Previous theoretical studies have proposed that the structure of an uncharged planar brush immersed in a chemically similar polymer melt, being among the simplest brush models, is determined by two main parameters: the grafting density *σ* and the ratio between the graft length *N* and the length of the free chains *P* in the melt [10,11,12,15,16,17]. Depending on the choice of these parameters, there are various regimes for the brush structure, from “mushrooms” under sparse grafting to “dry brush” under dense grafting [10,11,16,17]. For example, the excluded volume interactions under dense grafting may result in a strong stretching of the grafts from the surface and an almost complete expulsion of the free chains in the melt from the grafted layer. It has been shown that the dependence of the height *H* of the “dry” brush on *N* and *σ* is given by *H*~*Nσ* (omitting numerical prefactors) [10,11,16]. On the one hand, the brush height simply increases proportionately to increases in either *N* or *σ*, and no deviation from the strongly stretched graft’s conformation should occur. On the other hand, this result implies that the brush height does not change at different *N* and *σ* values, provided that their product *Nσ* is fixed. This commonality of brush behavior in a specific regime is a baseline for achieving a controllable design for the grafted layer with the required structure.

In our previous studies, the unusual behavior of the grafted lactide chains was observed by using atomistic molecular dynamics (MD) simulation when attempting to reproduce the known influence of the grafting density on the grafted layer structures [18,19]. Despite their dipolar nature, the lactide chains, being aliphatic ester chains, may initially seem to be the conventional grafts that were considered earlier [10,11,12,15,16,17]. A separation of the grafted lactide chains into two populations was observed in the melt of the chemically similar polymer, although only a strong stretching of the grafts was expected [18,19]. Importantly, the segregation was found to be particularly pronounced in the “dry brush” regime [19]. Only a fraction of the grafts was stretched from the surface, and, in contrast to the previous results, the other grafts were backfolded to the surface and adopted a loop-like conformation [10,11,16]. The Scheutjens–Fleer self-consistent field (SF–SCF) numerical calculations, the theoretical arguments based on a simple two-state model, and the atomistic MD simulations allowed us to confirm that the key factor leading to the backfolding of the lactide chains grafted via similar ends is related to dipole–dipole interactions between the longitudinal dipoles of the grafts [20]. While the performed MD simulations have provided valuable insights into the structure of the grafted lactide chains, a missing factor was that they were restricted to consideration of grafts with a fixed length. It is therefore of great importance to examine how the structure of the grafted lactide chains depends on the graft length *N* in the case of the “dry brush” regime, where the segregation is fairly pronounced.

In the present study, we aim to fill this gap by addressing the influence of the length of grafted lactide chains on the structure of their layer in the “dry brush” regime that is immersed in a chemically similar polymer melt by using atomistic MD simulations. For this purpose, we took the calculations of the fraction of backfolded lactide chains depending on the graft length as our starting point. Then, the normal and lateral density profiles were analyzed in the systems under consideration. This analysis was complemented by the evaluation of the order parameter along with typical snapshots of the grafted layer. Additionally, the helicity of the grafts was examined with an autocorrelation function for the vectors that connect the chiral atoms within the chain’s backbone. The structure of the grafted layers in the “dry brush” regime was also studied for two systems by using different values for *N* and *σ* with the same product. Finally, we analyzed the sensitivity of the simulation results to the choice of the systems sizes and the force field employed.

## 2. Model and Simulation Method

**Figure 1:**
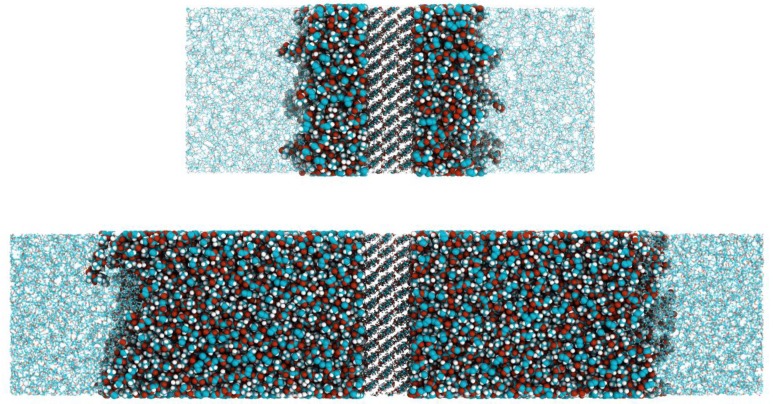
Snapshots of the polylactide-based nanocomposites filled with cellulose nanocrystals modified by the grafting of lactide chains with the length of 13 (top) and 50 (bottom) monomers at the grafting density *σ* = 1.76 nm^−2^ before a preliminary run. For visual clarity, cellulose nanocrystals chains in the middle of the nanocomposites are shown in the ball and stick representation, while grafted and free chains in the melt are drawn by van der Waals spheres and lines, respectively.

Following our previous simulations [18,19,20,21], we performed atomistic molecular dynamics simulations of polylactide-based nanocomposites filled with surface-modified cellulose nanocrystals (CNC). The simulated systems comprised 20 free lactide chains that surrounded the filler, with the length of the free chains equal to 150. Throughout this paper, the length of the chain denotes the number of monomers therein. The cellulose nanocrystals were periodic on the XY-plane and consisted of 36 cellulose chains with a length of 6 that were packed in three layers of 12 chains each. The CNC sizes in the XY-plane were 6.57 × 6.24 nm, with a thickness of 1.84 nm. All the primary hydroxyls on both the top and bottom surfaces of the cellulose nanocrystals were modified by grafting 72 lactide chains via the acid-terminated chain end, i.e., the highest possible degree of primary hydroxyls substitution was studied. Such a modification corresponded to the grafting density *σ* = 1.76 nm^−2^, at which the “dry brush” regime was observed [19]. Moreover, the segregation of the grafts into populations of backfolded and stretched chains was most pronounced at this *σ* in comparison with the other grafting densities [19,20]. Having fixed *σ*, the length of the grafted chains *N* was varied. Six lengths of the grafted chains, *N* = 13, 17, 22, 30, 40 and 50, were examined. Snapshots of the systems in the case of *N* = 13 and 50 are presented in Figure 1. Additionally, a grafted layer in the “dry brush” regime with the grafting density *σ* = 0.88 nm^−2^ and the graft length *N* = 60 was simulated. This system was compared with the one where the grafting density *σ* = 1.76 nm^−2^ and the graft length *N* = 30 in order to examine difference in the structure between the two grafted layers at a fixed product of *Nσ*. According to our previous estimate of the characteristic ratio [22], the Kuhn segment length *A* for the lactide chains is equal to approximately 1.6 nm. The contour length *L* of the grafts under consideration could be calculated as L=aN, where *a* = 0.38 nm was the contour length of the monomer in the graft. These calculations gave contour lengths *L* in the range 5–19 nm for the considered length of the grafts at *σ* = 1.76 nm^−2^. Thus, the number of Kuhn segments in the grafts at this *σ* varied from approximately 3 to 12 units, lying in different orders of magnitude. For the grafts where *σ* = 0.88 nm^−2^, the contour length and the number of Kuhn segments were equal to about 23 nm and 14 units, respectively. The chosen graft lengths were limited by the computational demands required to perform simulations of the corresponding composites. To the best of our knowledge, the considered grafts were among the longest ever studied by using atomistic molecular dynamics simulations. In the present study, the total number of atoms in the system with the grafted layer at *σ* = 1.76 nm^−2^ and *N* = 13 was about 53,000, while the composite contained about 100,000 atoms in the case of the grafted layer at *σ* = 1.76 nm^−2^ and *N* = 50. It should be noted that the impact of the choice of the system size on the obtained simulation results was additionally evaluated; see the Results and Discussion section.

In contrast to our previous studies, for the present work, the GAFF force field [23] was employed to describe the bonded and non-bonded interactions in the simulated composites [18,19,20,21]. The use of this force field for the simulations of lactide chains was validated in reference [22] on the basis of calculations for the chain’s flexibility and the glass transition temperature for the corresponding bulk system. The present study, however, was devoted to composites filled with surface modified cellulose nanocrystals. The PLAFF3 force field [24], specifically developed for lactide chains on the basis of the OPLS force field [25], has been previously used for these systems [18,19,20,21]. It was necessary to implement additional interaction parameters from the OPLS force field that described pristine and modified cellulose chains in order to simulate the required composites. Since these composites are heterogeneous, i.e., they contain both lactide and cellulose chains, we chose the GAFF force field, which enabled a complete description of the systems without the special combining of the interactions’ parameters. A comparison of the outcomes of simulations of composites performed using both the GAFF and PLAFF3 force fields previously showed the consistency of the obtained data, validating the use of these force fields [26]. Nevertheless, we additionally compared the sensitivity of the simulation results presented to the choice of force field, since new structural features of the composites were revealed; see the Results and Discussion section. As recommended by the GAFF force field developers [23], we utilized the partial atomic charges that were calculated by the Hartree−Fock (HF) approach with 6-31G* basis set of wave functions and the RESP method of charges evaluation using the Gaussian 09 software [27] in our previous study [26]. Additionally, the systems considered without partial charges were simulated as “reference” models, reproducing the known behavior of the uncharged conventional polymer brushes [19].

In common with our previous studies [18,19,20,21], the initial stage of the simulations included the surrounding of the modified CNC with pre-equilibrated free lactide chains. Then, compression was performed for 5 ns at a temperature of 600 K and a pressure of 50 bar along the Z-axis normal to the filler surface. After the compression, the obtained systems were simulated for 1 µs at a temperature of 600 K and at a pressure of 1 bar normal to the CNC surface. The choice of the simulation temperature of 600 K stemmed from the need to study the grafted layer immersed in the melt, as well as the need to accelerate the equilibration [22,28]. It is worth mentioning that the mean-square end-to-end distance *H_end-to-end_* of the examined grafts reached a constant value during the first 0.3 µs of the simulations; see Figure S1 in Supplementary Material (SM). This part of the simulation trajectory was defined as a preliminary run. In turn, the remaining 0.7 µs of the trajectory was used as a production run for the structural analysis of the systems under study.

The GROMACS 5.1.4 was employed to perform the simulations [29]. The stability of the CNC was maintained by the position restraints algorithm [30], and the motion equations were integrated with a time step of 1 fs [31,32]. Temperature and pressure were coupled using the Berendsen thermostat and barostat with time constants of 0.1 ps and 1 ps, respectively [33,34]. The particle-mesh Ewald (PME) method was used to handle electrostatics [35]. Both the Lennard-Jones and electrostatics interactions were truncated at 1 nm [36,37,38]. Simulation trajectories were visualized by the VMD software [39]. The structure of the systems examined was analyzed using in-house Python scripts written with the aid of the MDAnalysis library [40,41].

## 3. Results

### 3.1. Fraction of Backfolded Chains

**Figure 2:**
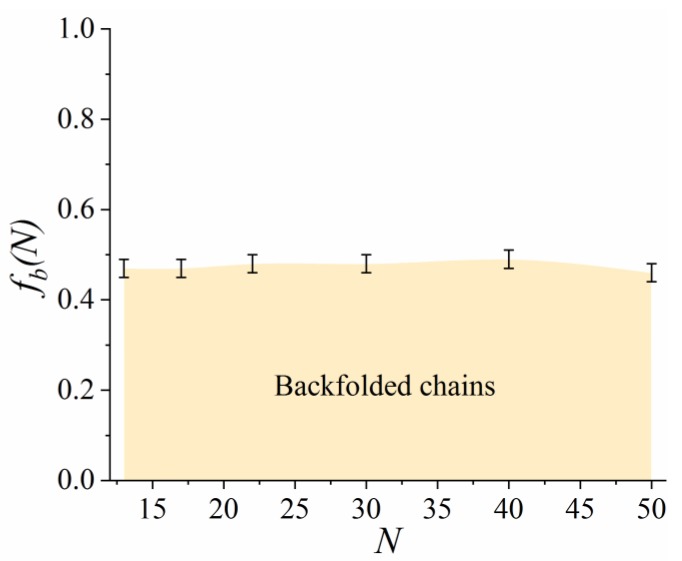
Dependence of the fraction of backfolded lactide chains *f_b_* on the graft length *N.*

One of the characteristic structural features of the grafted lactide chains is their backfolding to the filler surface [18,19,20]. Therefore, we first discuss how the graft length *N* influences the fraction of the backfolded lactide chains $f_{b}$ within the grafted layer. For this analysis, the distribution of the position of the center of mass in the grafted chains in the simulated systems was calculated. The fraction of the backfolded chains $f_{b}$ was estimated as the area of the first maximum in the distribution, as illustrated in Figure S2. Figure 2 shows the $f_{b}(N)$ dependence obtained.

The fraction of the backfolded grafts remained approximately half of the chains, which within the margin of error for all the chain lengths considered. The fact that almost no influence of *N* on *f_b_* was observed is in line with the previous numerical self-consistent field calculations in the case of grafted dipolar chains [20].

Since the fraction of the backfolded chains was found to be almost constant, one can assume that the structure of the grafted layer did not qualitatively change with changes of the chain length *N*. In the following sections, we focus on structural details in the simulated systems to verify this assumption.

### 3.2. Normal and Lateral Density Profiles of the Grafted Chains

**Figure 3:**
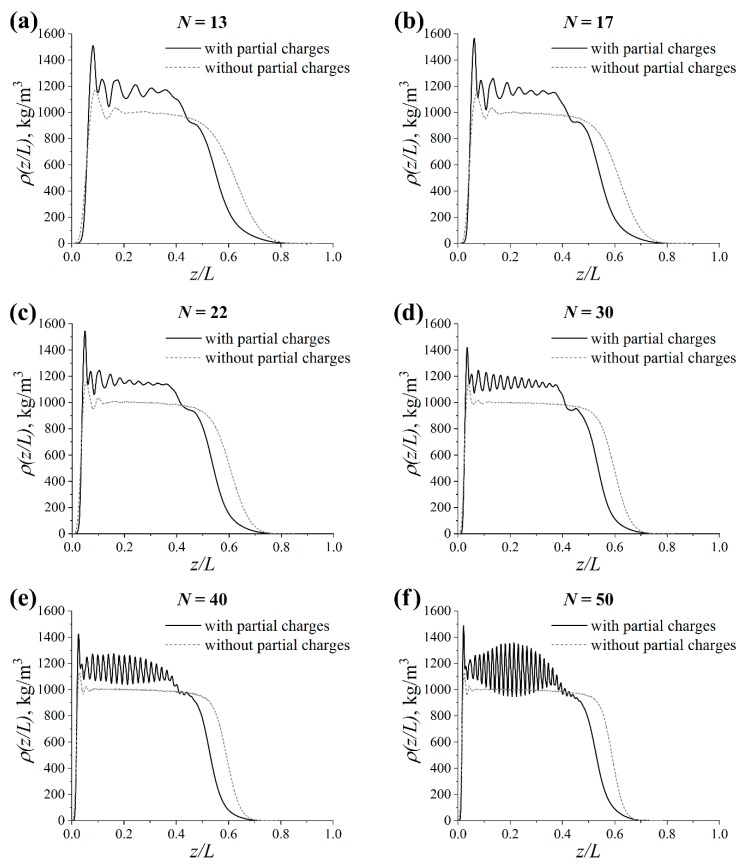
Normal density profiles of the grafted chains *ρ*(*z/L*) related to the cellulose nanocrystals (CNC) surface at different graft lengths *N* in the systems with and without partial charges: (**a**) *N* = 13, (**b**) *N* = 17, (**c**) *N* = 22, (**d**) *N* = 30, (**e**) *N* = 40 and (**f**) *N* = 50. *L* is the graft contour length.

To describe the structure of the grafted layer, the normal density profiles of the grafted chains *ρ* related to the CNC surface at different chain lengths *N* for the systems with and without partial charges are presented in Figure 3. For simplicity, the profiles are plotted with respect to the normalized distance *z/L* along the *z*-axis from the CNC surface, where *L* is the contour length of the graft. In addition, the profiles that were normalized with the Kuhn segment length for the lactide chains are presented in Figure S3.

Figure 3 shows that the *ρ*(*z/L*) dependence for the systems under study exhibited two maxima near the CNC surface. These maxima could be attributed to the presence of a short-range order similar to that in the case of hard spheres near a repulsive wall [42,43,44].

At the same time, an extended plateau could also be observed in the density profiles for the systems without partial charges; see Figure 3. This means that the grafts were strongly stretched from the surface [45]. Since the choice of the grafting density and the lengths of grafted and free chains corresponded to the “dry brush” regime [16], this result was to be expected. The form of the density profile became step-like as the graft length *N* increased. Overall, no qualitative change in the *ρ*(*z/L*) dependence was established at the various considered values for *N*. This indicates the generality of the influence of the chain length on the structure of the grafted layer, which is in line with previously known results [16,46].

By contrast, in the systems with partial charges, the graft length had a more complex effect on the structure of the grafted layer. In line with our previous observations [19], the average density of the grafted chains near the CNC surface in systems with partial charges was higher than that in the case of systems without partial charges, regardless of the graft length *N*; see Figure 3. One can understand this by the effect of the grafts backfolding to the CNC surface, leading to an increase in the local density of the grafted layer [19]. Surprisingly, pronounced oscillations were observed far from the filler surface. This could not solely be explained by the presence of the surface since both the number of maxima and their frequency increased as the graft length *N* increased. Indeed, the oscillations at *N* = 30 became dramatically enhanced as compared to the case of *N* < 30, suggesting a qualitative change in the structure of the grafted layer; see Figure 3a–c. The chain length *N* = 30 corresponded to approximately 7 Kuhn segments. Note that the further increase of *N* from 30 to 50 did not lead to qualitatively new results: Only the amplitude of the oscillations increased; see Figure 3d–f. It is possible that a special packing with an ordering of monomers was present in the systems, starting with the graft lengths of about 10 Kuhn segments.

**Figure 4:**
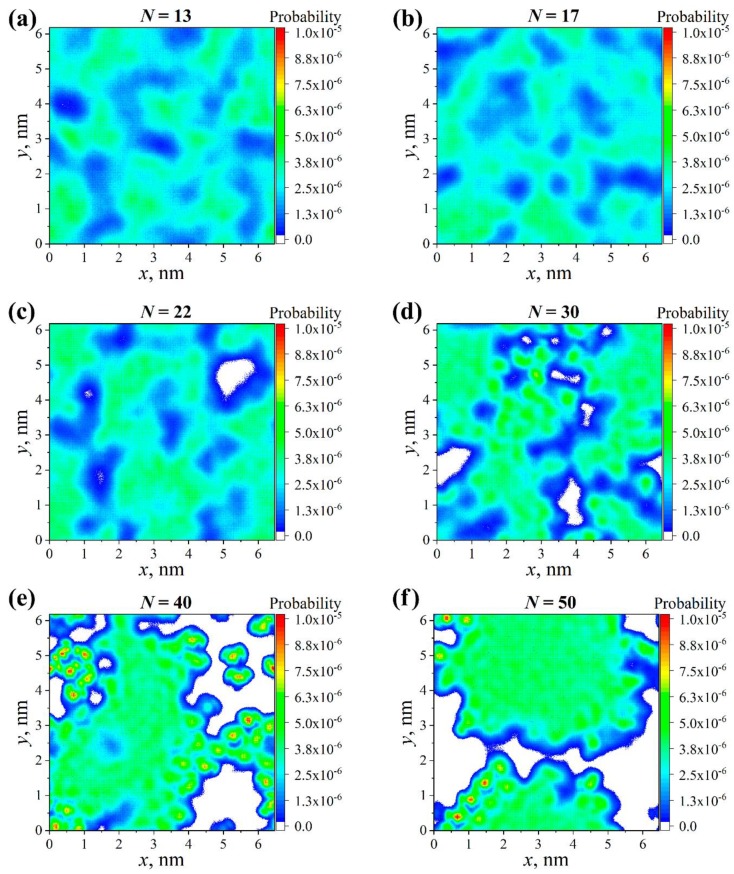
Lateral density profiles of the backfolded grafted chains at different graft lengths *N* for systems with partial charges: (**a**) *N* = 13, (**b**) *N* = 17, (**c**) *N* = 22, (**d**) *N* = 30, (**e**) *N* = 40 and (**f**) *N* = 50.

To complement the analysis of the normal density profiles, we additionally evaluated the influence of the graft lengths on the lateral packing and the ordering of both backfolded and stretched grafts in the systems with partial charges. The lateral density profile of the backfolded and stretched grafts within a thin layer at *z* = 1–1.5 nm projected on the CNC surface (XY-plane) was evaluated over the last 100 ns of the simulations, following the studies in references [47,48]. We noted that a different choice of thickness and position of the thin layer did not alter the results significantly. The color in the lateral density profile (see Figure 4) denotes the probability of finding an atom of a graft with certain *x*- and *y*-coordinates: A “warmer” color indicates a higher probability. Therefore, this profile allowed us to determine the mutual location of the grafts in the plane of the filler surface.

Figure 4a shows that the backfolded grafts where *N* = 13 were homogeneously distributed within the grafted layer. An increase to the graft length *N* led to a thickening of the regions corresponding to the density of the backfolded grafts; see Figure 4b–e. In other words, the grafted layer became less homogeneous than in the case where the graft length *N* = 13. As seen in Figure 4f, it is evident that the grafted layer was fairly inhomogeneous, with the backfolded chains forming a separate region. Moreover, one can observe the presence of a somehow ordered group of chains following the line that connects two points: (0.5 and 0.0 nm) and (2 and 2 nm). It should be noted that the lateral density profiles of the chains stretched from the surface brought similar details on the distribution and the ordering of the grafts; see Figure S5. Thus, an increase to the grafts’ lengths resulted in a lateral inhomogeneity and an ordering of the backfolded and stretched grafted chains.

Overall, our assumption on the commonality of the influence of graft length on the structure of the grafted layer turned out to be valid only for the systems without partial charges. It was evident that the structure of the grafted layer in the systems with partial charges could be qualitatively different depending on the graft length *N*. Importantly, both the normal and lateral density profiles indicated that a change to the order occurs upon increasing *N* in the systems with partial charges.

### 3.3. Order Parameter within the Grafted Layer

Let us now turn to analyzing the order in the simulated systems. To this end, the second Legendre polynomial *P*_2_(*z*/*L*) was calculated as [49]:

(1) $$P_{2}(z/L)=\frac{1}{2}\left(3\langle \cos^{2}\theta (z/L)\rangle -1\right)$$

where θ(z/L) is the angle between the normal to the CNC surface and the vector along the graft monomer at the normalized distance z/L along the *z*-axis from the surface.

**Figure 5:**
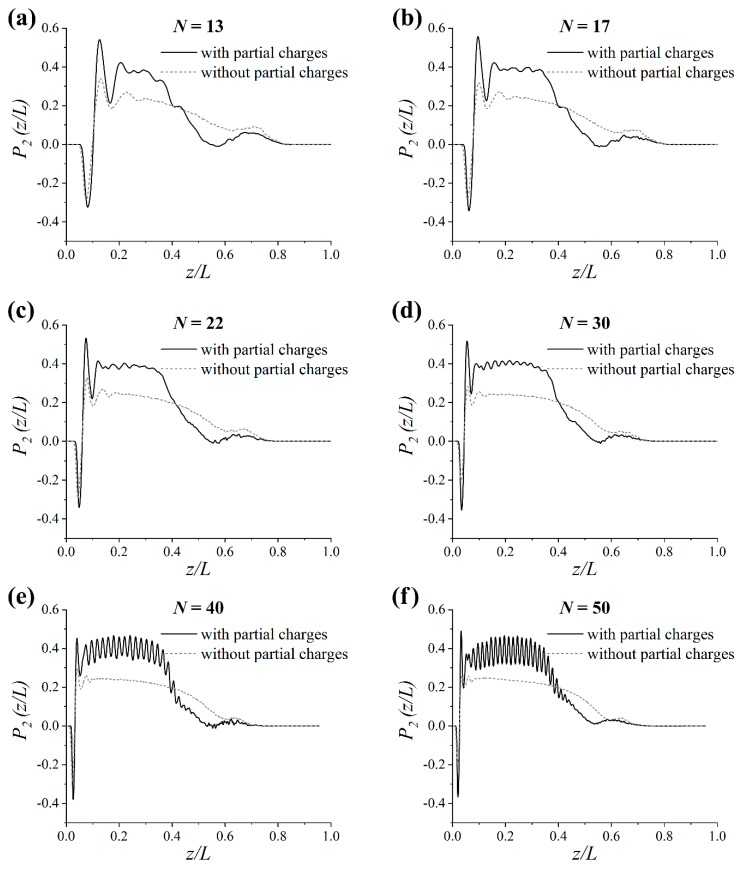
Order parameter $P_{2}(z/L)$ for the grafts’ monomers as a function of their normalized distance from the filler surface z/L at different chain lengths *N* for the systems with and without partial charges: (**a**) *N* = 13, (**b**) *N* = 17, (**c**) *N* = 22, (**d**) *N* = 30, (**e**) *N* = 40 and (**f**) *N* = 50. *L* is the graft contour length.

As illustrated in Figure 5, the order parameter $P_{2}(z/L)$ was perturbed near the CNC surface in the systems under study. The negative peak may indicate the flattening of the first monomer of the grafted chain on the surface, while the pronounced positive peak can be ascribed to the second monomer with a large degree of alignment normal to the surface. These results explain two maxima attributed to the presence of a short-range order near the CNC surface in Figure 3.

A smooth decrease of $P_{2}(z/L)$ from the value of about 0.2 to zero value occurred in the systems without partial charges for larger distances from the surface; see Figure 5. This meant that the grafts were somewhat stretched from the surface. Similar values for the order parameter were previously reported for stretched grafted chains in references [49,50]. Thus, the order parameter looked almost identical at all chain lengths considered in the systems without partial charges.

In the systems with partial charges the order parameter reached values, on average, two times greater compared to the systems without partial charges, indicating a much greater degree of alignment of the grafts normal to the surface. For *N* = 13, the initial increase of $P_{2}(z/L)$ was followed by a gradual decrease up to distances z/L ≈ 0.5; see Figure 5a. As follows from Figure 5b,c for *N* = 13 and 22, the dependence of the order parameter exhibited an extended plateau around $P_{2}(z/L)$ ≈ 0.4 with irregular maxima. In contrast, an extended plateau with fairly regular oscillations around $P_{2}(z/L)$ ≈ 0.4 was clearly seen in the case of grafts with *N* = 30; see Figure 5d. Thus, a considerable enhancement of the order in the systems was obtained due to the increase in graft length. This order became even more pronounced when the chain length value was increased from 30 to 50; see Figure 5e,f. It should be noted that a correlation between the peaks positions in the density profile and the order parameter dependence is present in Figure 3 and Figure 5. The distance between the peaks was approximately equal to the monomer size.

Therefore, analysis of the order in the systems revealed the presence of a considerably higher degree of graft alignment relative to the surface in systems with partial charges, as compared to those without partial charges. Moreover, the ordering with strictly regular monomer positions that was suggested from the analysis of the density profiles was confirmed. Thus, it is of great interest to discuss exactly how the packaging of the grafts changed while increasing the chain length *N*.

### 3.4. Visual Analysis of the Graft’s Conformation

**Figure 6:**
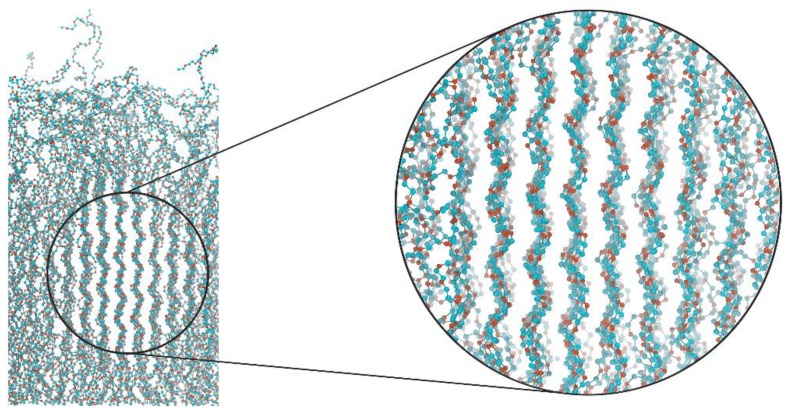
Typical snapshots of the grafted layer in the case of *N* = 50 for the system with partial charges. The inset on the right side shows a magnified region, with the grafts adopting a conformation close to a helix.

**Figure 7:**
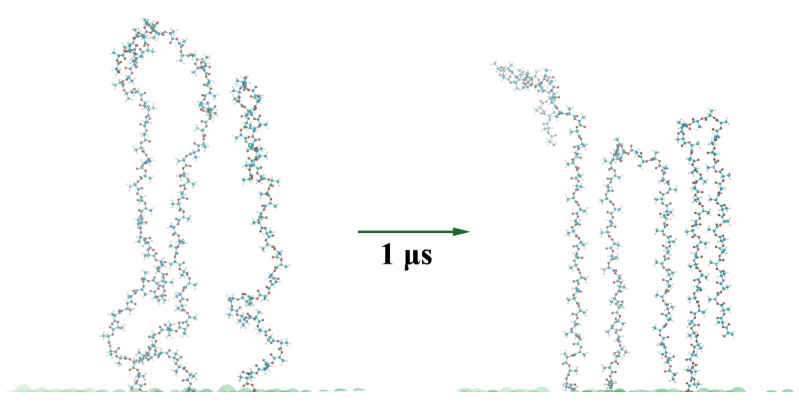
Configuration of three chains within the grafted layer where the graft length *N* = 50 at the initial moment of the simulations (**left**) and after the microsecond-long simulations (**right**). The green hemispheres show the grafting surface at the bottom of the figure.

To understand a possible way of the ordering, a visual analysis of typical snapshots of the grafted layer where *N* = 50 was performed. In this case, the strongest oscillations were observed on the normal density profiles *ρ*(*z/L*) (Figure 3) and the order parameter dependence *P_2_*(*z/L*) (Figure 5) in comparison with the other grafted layers. Figure 6 shows the existence of an ordered region within the system, which may have been responsible for the presence of the pronounced oscillations observed in *ρ*(*z/L*) and *P_2_*(*z/L*) (Figure 3 and Figure 5). Importantly, one can also notice that the grafts adopted a conformation close to a helix. A visualization of the simulation trajectories (see Figure 7) for the grafts in the considered systems additionally clarified that both the stretched and backfolded chains had some helix-like fragments along their contour. In particular, helix-like structures were observed for almost the half of the contour of a stretched chain, by which it was grafted to the filler surface. At the same time, both halves of the contour of a backfolded chain may have simultaneously looked like helixes. The backfolded chain had almost no helicity at the kink point. Thus, the visual analysis performed enabled us to assume that the grafts adopted a helical conformation.

### 3.5. Helicity of the Grafted Chains

**Figure 8:**
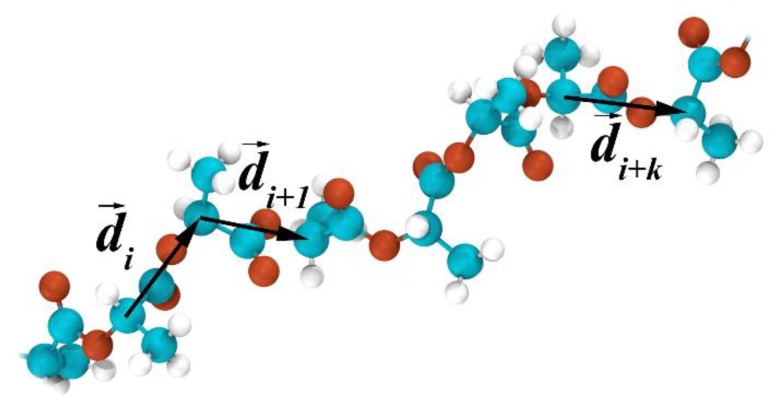
Schematic illustration that shows the choice of the unit vectors to calculate the autocorrelation function C(k) for the grafts.

In order to validate the proposed assumption and to examine whether helical conformations were formed, the helicity of the grafts was analyzed. It was already known that the presence of helical conformations and the structure of helices in polymer chains can be quantified using an autocorrelation function C(k) of the vectors along the chain’s backbone [51,52]. The autocorrelation function C(k) for the considered grafted chains was estimated as [51]:

(2) $$C(k)=\frac{1}{N-k}\sum_{i=1}^{N-k}\left|({\vec{d}}_{i}{\vec{d}}_{i+k})\right|$$

where *N* is the number of monomers within a graft and *k* is the number of monomers between the unit vectors ${\vec{d}}_{i}$ and ${\vec{d}}_{i+k}$ connecting the chiral atoms along the graft backbone; see Figure 8.

**Figure 9:**
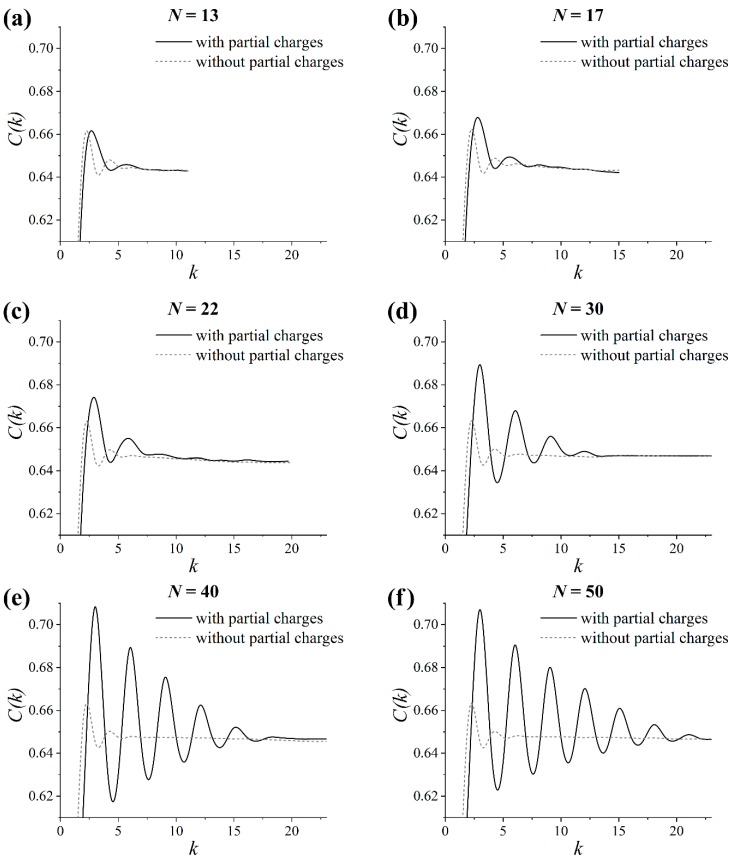
Autocorrelation function C(k) for the grafts with different chain lengths *N* in the systems with and without partial charges: (**a**) *N* = 13, (**b**) *N* = 17, (**c**) *N* = 22, (**d**) *N* = 30, (**e**) *N* = 40 and (**f**) *N* = 50.

One can see from Figure 9 that there were two small maxima at *k* ≈ 2.3 and *k* ≈ 4.3 in the C(k) plots for the systems without partial charges. Therefore, only a short-range correlation of the monomers’ direction was observed along the contour of the grafts, regardless of their length in these systems.

This was also the case for the grafts with *N* < 30 in the systems with partial charges; see Figure 9a–c. However, increasing the graft length value up to 30 enhanced the oscillations of C(k) and led to an increase in the number of peaks, thereby suggesting the formation of helical fragments in the system; see Figure 9d. In the case of the longer grafts (i.e., where *N* > 30), the results were qualitatively similar to the grafts with *N* = 30; see Figure 9d–f. Quantitatively, the greater the chain length considered, the longer the helical fragments could be observed for *N* > 30. Thus, the analysis of the autocorrelation function C(k) confirmed our initial assumption and showed that the grafted lactide chains adopted a helical conformation.

From an experimental standpoint, lactide chains can form ordered structures upon crystallization [53]. The α crystal is the most common polymorph obtained by crystallization from the melt state at temperatures above approximately 390 K [54]. The ideal α crystal consists of two antiparallel chain fragments in the 10_3_ helical conformation that are packed in an orthorhombic unit cell [55,56,57]. In other words, the fragment of a lactide chain in the α crystal represents the sequence of 10 monomers in three helical turns that is equal to approximately 3.3 monomers per turn. Therefore, it was important to check whether the graft’s helical conformation is similar to those for the fragment of the lactide chain in the α crystal.

**Figure 10:**
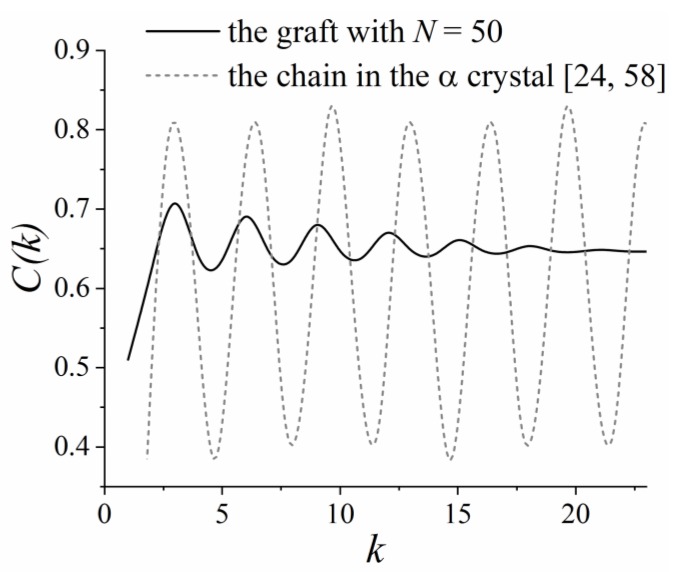
Autocorrelation function C(k) for the simulated grafted chains with length *N* = 50 and for the lactide chains in the α crystal, the structure of which was reported in references [24,58].

To this end, the α crystal of polylactide, which was constructed in reference [24] on the basis of the X-ray diffraction data reported by Sasaki and Asakura [58], was additionally analyzed. Figure 10 shows the autocorrelation function C(k) for the lactide chains in the α crystal in comparison with that for the simulated grafted chains with length *N* = 50, where the longest helical fragments were formed among the considered grafts. It could be seen that the grafts formed a helical conformation with about 3 monomers per turn, while the helical turn included about 3.3 monomers in the α crystal. Thus, a good agreement between the simulation results and the experimental data [58] for the α crystal was found. This result was also valid for the grafts with length *N* = 30 and 40 since the positions of the peaks of the C(k) dependence in these cases were almost identical to that for *N* = 50; see Figure 9d–f. Note that the amplitude of the peaks in the C(k) plot (Figure 10) for the grafts was lower than for the crystalline chains. This difference can be attributed to the fact that all the grafts’ fragments, both ordered and disordered, were considered in the calculations of C(k).

Overall, the helicity analysis confirmed that a long-range order may have been present in the layer of the grafted lactide chains longer than 30 monomers (about 10 Kuhn segments). The ordering was very similar, albeit much weaker, to that of the α crystal of polylactide; the grafts adopted a helical conformation close to that of the α-crystalline chains. Some helical fragments of the grafts were aligned antiparallel to each other due to the backfolding of the lactide chains within the grafted layer. In our opinion, the formation of such a structure may be regarded as the initial stage of crystallization of the grafted layer, a stage that was induced by the grafting of the lactide chains and driven by dipole–dipole interactions.

### 3.6. Influence of Grafting Density on the Structure of the Grafted Layer

**Figure 11:**
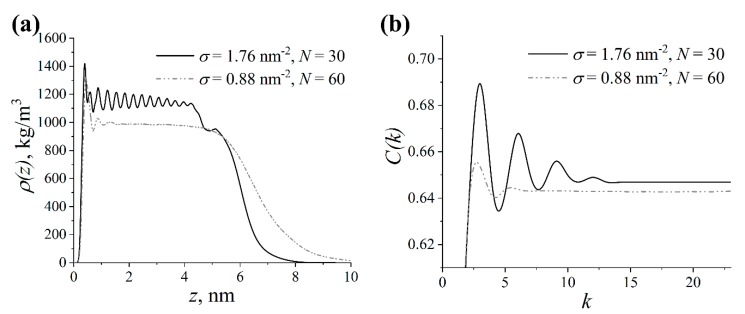
(**a**) Density profile ρ(z) and (**b**) autocorrelation function C(k) in the systems with partial charges, in which the graft length and the grafting density (*N*, *σ)* are equal to (30, 1.76 nm^−2^) and (60, 0.88 nm^−2^).

As mentioned in the Introduction, the structure of a brush in the “dry brush” regime does not qualitatively change when *N* and *σ* are varied but their product remains constant. To verify this rule for the grafted lactide chains under study, we compared the structure of the grafted layer in two systems with partial charges, in which (*N*, *σ)* were equal to (30, 1.76 nm^−2^) and (60, 0.88 nm^−2^). The density profile ρ(z) and the autocorrelation function C(k) obtained for these systems are presented in Figure 11.

Figure 11 shows that the density profiles ρ(z) and autocorrelation functions C(k) for the systems considered were qualitatively different, highlighting that we did not reproduce similar behavior of the structure of grafted layer at a constant value for *Nσ*. While ρ(z) exhibited pronounced oscillations for the grafted layer at *N* = 30 and *σ* = 1.76 nm^−2^, a smooth plateau region at relatively lower density values was seen for the other layer at *N* = 60 and *σ* = 0.88 nm^−2^; see Figure 11a. To put it simply, no structural ordering was observed in the latter system. The calculation of the thickness *H* of the grafted layer as the first moment of the density profile yielded values of 3.1 ± 0.1 nm and 3.6 ± 0.1 nm for the former and latter system, respectively. Comparing the autocorrelation function C(k) in Figure 11b, one could identify that, in contrast to the other chains at *N* = 30 and *σ* = 1.76 nm^−2^, the lactide chains with length *N* = 60 grafted at *σ* = 0.88 nm^−2^ had only a short-range correlation between the monomers along their contour. In summary, the data obtained contradict the existing theoretical results on the structure of polymer brushes in a “dry brush” regime immersed in a chemically similar melt. Namely, the dependence of brush height and structure on the combination *Nσ*, which holds for regular non-polar brushes, is no longer valid for the brushes made of polar lactide chains. In general, it is of great interest to estimate the critical grafting density, beyond which there is the ordered structure, as well as the corresponding scaling laws for the brush height, depending on *σ* and *N.* However, these tasks are very demanding in terms of the computational resources required for the atomistic simulations and will be additionally addressed in our further studies.

The results obtained raise several additional questions that need to be addressed. One question that merits attention is related to the temperatures *T*, at which our simulations were conducted. One can argue that the observed structural order at *T* = 600 K was counterintuitive, since the experimental melting temperature of the polylactide α crystal *T_m_* ≈ 460 K [59]. Note, however, that this melting temperature was measured for free polylactide chains, while our results were related to grafted chains. For grafted chains, it is known that grafting may result in a significant reduction to the dynamics of the grafts due to the following factors: Firstly, one of their ends is fixed, and secondly they are confined within the grafted layer [60,61]. Moreover, the glass transition temperature of the grafted chains may be higher than that of the free ones [60]. Therefore, it can be assumed that the structural order of the observed lactide chains was related to the effect of the chains grafting, which prevented the grafts from melting at temperature *T* = 600 K.

Other important points which should be discussed are related to the effect of the choice of force field and to the finite size of the simulated systems. To address the sensitivity of the simulation results to both the force field and the system sizes used, a system with larger sizes was additionally simulated using the GAFF and PLAFF3 force fields. Lactide chains with length *N* = 50 grafted at *σ* = 1.76 nm^−2^ were examined. For this system, the sizes of the grafting surface and the number of grafts were increased fourfold. The number of free chains in the melt was increased eightfold to 160. Overall, the total number of atoms in the system was changed from about 100,000 to 511,000. We repeated the simulation protocol used in the present study, with the exception that the simulations were run for 550 ns instead of 1 µs due to the drastically increased computational expense involved. In the case of the GAFF force field, almost no change in the C(k) was observed upon increasing the system size. The autocorrelation functions C(k) for the grafts in the systems with reference sizes and larger sizes were found to be fairly similar; see Figure S6 in SM. Thus, a good consistency of the data on the graft’s helicity was obtained across different system sizes. When employing the PLAFF3 force field instead of GAFF, the number of monomers per helical turn changed from about 3 to 2. The outcomes of the simulations with the GAFF and PLAFF3 force fields were quantitatively different. The GAFF force field reproduced the number of monomers per helical turn slightly better than the PLAFF3 force field. Nevertheless, both GAFF and PLAFF3 force fields resulted in a qualitatively similar helicity of the grafts. Therefore, it was shown that the main results of the present study were not sensitive to the system sizes and the force field used.

## 4. Conclusions

We examined the influence of the length of grafted lactide chains *N* on the structure of a grafted layer immersed in a melt of chemically similar polymer by using microsecond atomistic molecular dynamics simulations. The case of the “dry brush” regime at the grafting density *σ* = 1.76 nm^−2^ (the highest possible substitution of the primary hydroxyls of the filler surface) was considered. The presence of a rather pronounced segregation of the chains into populations of backfolded and stretched grafts due to dipole–dipole interactions has been previously found at this grafting density, when lactide chains were grafted to a filler surface using similar chain ends [19,20].

It was shown that the fraction of the backfolded chains did not change with the graft lengths *N* being considered. Unexpectedly, the normal and lateral density profiles indicated the formation of a qualitatively new structure of the grafted layer upon the increase of the graft length *N* to about 10 Kuhn segments. The possibility of an ordered packing of monomers of the backfolded and stretched grafts was suggested. Moreover, it turned out that the grafted layer became laterally inhomogeneous. The presence of strictly regular monomer positions was revealed by the evaluation of the order parameter. A visual analysis of typical snapshots of the grafted chains considered enabled us to assume that the stretched and backfolded grafts adopted a helical conformation. In turn, an analysis of the graft’s helicity confirmed the formation of helical fragments within the grafted layer. A good agreement was established between the simulation results and the experimental data for the α crystal of lactide chains on the number of monomers per helical turn. Thus, the grafting of lactide chains induced both the backfolding of the grafts and their helical ordering, which can be considered as the initial stage of crystallization of the grafted layer.

At the same time, we compared the structure of systems with a fixed product *Nσ*, where (*N*, *σ)* were equal to (30, 1.76 nm^−2^) or (60, 0.88 nm^−2^). In contrast to the known theoretical picture, though both systems corresponded to the “dry brush” regime and a similar structure was expected, no structural ordering was seen for the latter case at *N = 60* and *σ* = 0.88 nm^−2^. It should be noted that no qualitative change in the structure of the grafted layer was obtained regardless of the *N* in the systems simulated without partial charges, suggesting the crucial role of dipole–dipole interactions for the possible structural features of the grafted lactide chains.

In conclusion, the presented analysis demonstrates that the grafting of lactide chains via similar chain ends may induce not only their segregation but also their structural ordering. This highlights how rich the structure of the grafted lactide chains can be. We hope that our results will inspire future experimental studies of the structure of grafted lactide chains in such systems, as well as the development of new theories that revisit the polymer brushes made of grafted dipolar chains immersed in a melt.

## Supplementary Materials

The following are available online at https://www.mdpi.com/2073-4360/11/12/2056/s1, Figure S1: Time dependence of the mean square end-to-end distance *H_end-to-end_* for the grafted lactide chains with different lengths *N*. The vertical dash line shows the preliminary simulations time of 300 ns. Figure S2: Probability density *p(R_com_)* to find the center-of-mass of the graft at the distance *R_com_* relative to the filler surface in the systems with different graft lengths *N*. Figure S3: Normal density profiles of the grafted chains *ρ(z/A)* related to the CNC surface at different graft lengths *N* in the systems with and without partial charges. Figure S4: Order parameter *P_2_(z/A)* for the grafts’ monomers as a function of their normalized distance from the filler surface *z/A* at different chain lengths *N* for the systems with and without partial charges. Figure S5: Lateral density profiles of the grafted chains stretched from the filler surface at different chain lengths *N* for the systems with partial charges. Figure S6: Autocorrelation function C(k) for the grafts with the chain length *N* = 50 in the systems with reference sizes (used in the present paper) and larger sizes simulated in the GAFF and PLAFF force fields.
