# Supplementary material for: Grafting-Induced Structural Ordering of Lactide Chains
Source: Polymers (Basel). 2019 Dec 11;11(12):2056. doi: 10.3390/polym11122056 (PMC6961058; doi:10.3390/polym11122056)
Supplement: Supplementary file 1 [file polymers-11-02056-s001.pdf]

Supplementary Material for

Grafting-Induced Structural Ordering of Lactide Chains

Artyom D. Glova [1], Sofya D. Melnikova [2], Anna A. Mercurieva [1], Sergey V. Larin [1] and Sergey V. Lyulin [1,*]

[1] Institute of Macromolecular Compounds, Russian Academy of Sciences, Bolshoj pr. 31 (V.O.), 199004 St. Petersburg, Russia; glova@imc.macro.ru (A.D.G.); anna@macro.ru (A.A.M.); selarin@macro.ru (S.V.L.)
[2] Institute of Physics, Nanotechnology and Telecommunications, Peter the Great St. Petersburg Polytechnic University, Polytechnicheskaya st. 29, 195251 St. Petersburg, Russia; sofya_m1@mail.ru
[*] Correspondence: s.v.lyulin@gmail.com; Tel.: +7-812-323-0216

1. Mean square end-to-end distance of grafted lactide chains

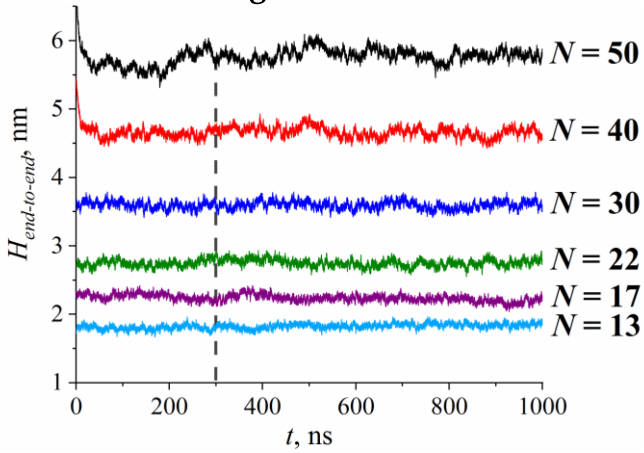

Figure S1. Time dependence of the mean square end-to-end distance $H_{end\text{-}to\text{-}end}$ for the grafted lactide chains with different lengths N. The vertical dash line shows the preliminary simulations time of 300 ns. Note that the decrease of $H_{end\text{-}to\text{-}end}$ for the grafts with $N = 40$ and 50 can be ascribed with the fact that the grafted chains were vertically oriented at the very beginning of the simulations (before compression); it takes some time for the grafts to stretch, since their length is higher than the one of the other grafts.

2. Distribution of center-of-mass position of grafted chains

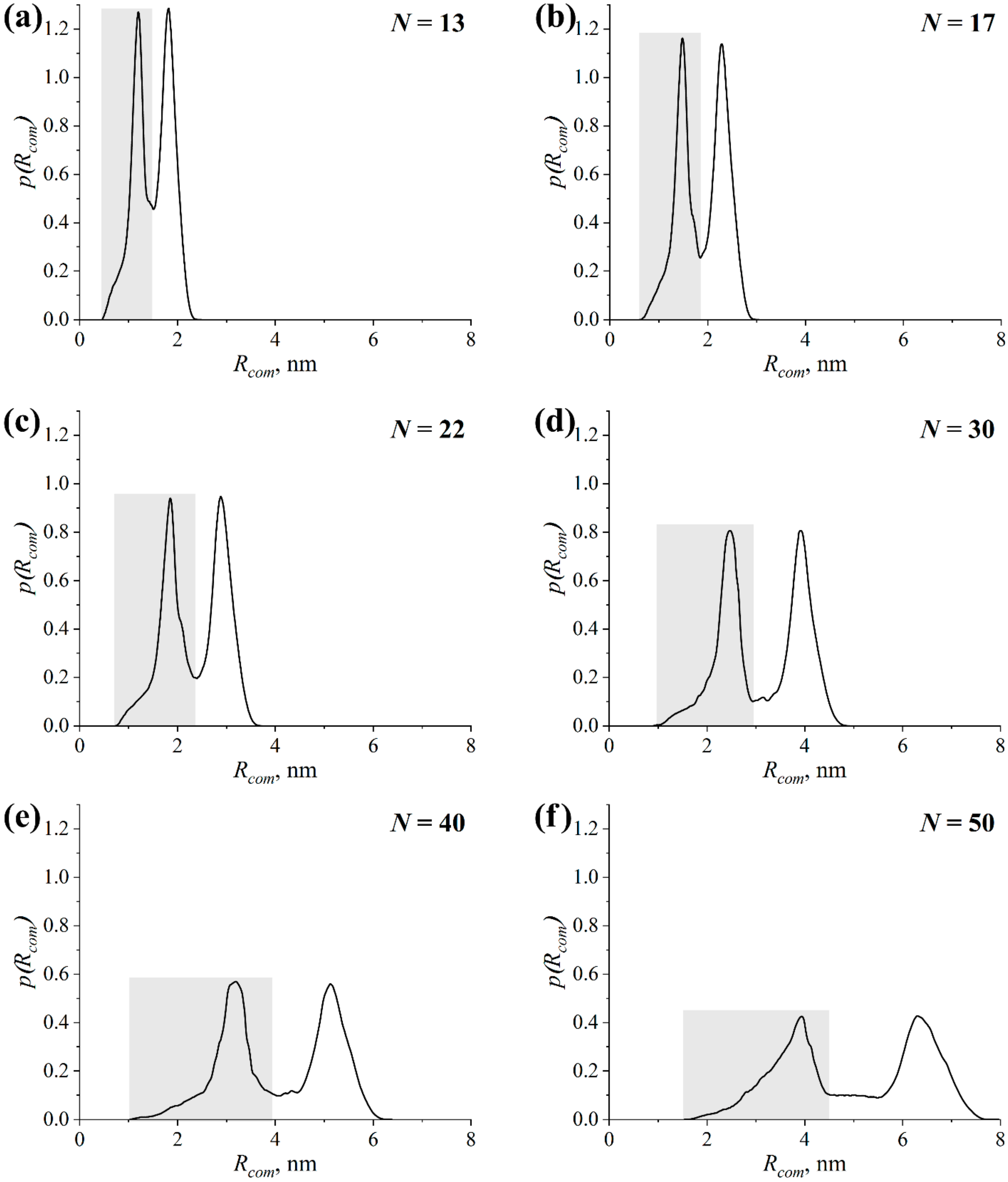

Figure S2. Probability density $p(R_{com})$ to find the center-of-mass of the graft at the distance R_{com} relative to the filler surface in the systems with different graft's length N. Area colored in gray indicates the maximum corresponding to the fraction of the backfolded chains.

3. Normal density profiles of the grafted chains

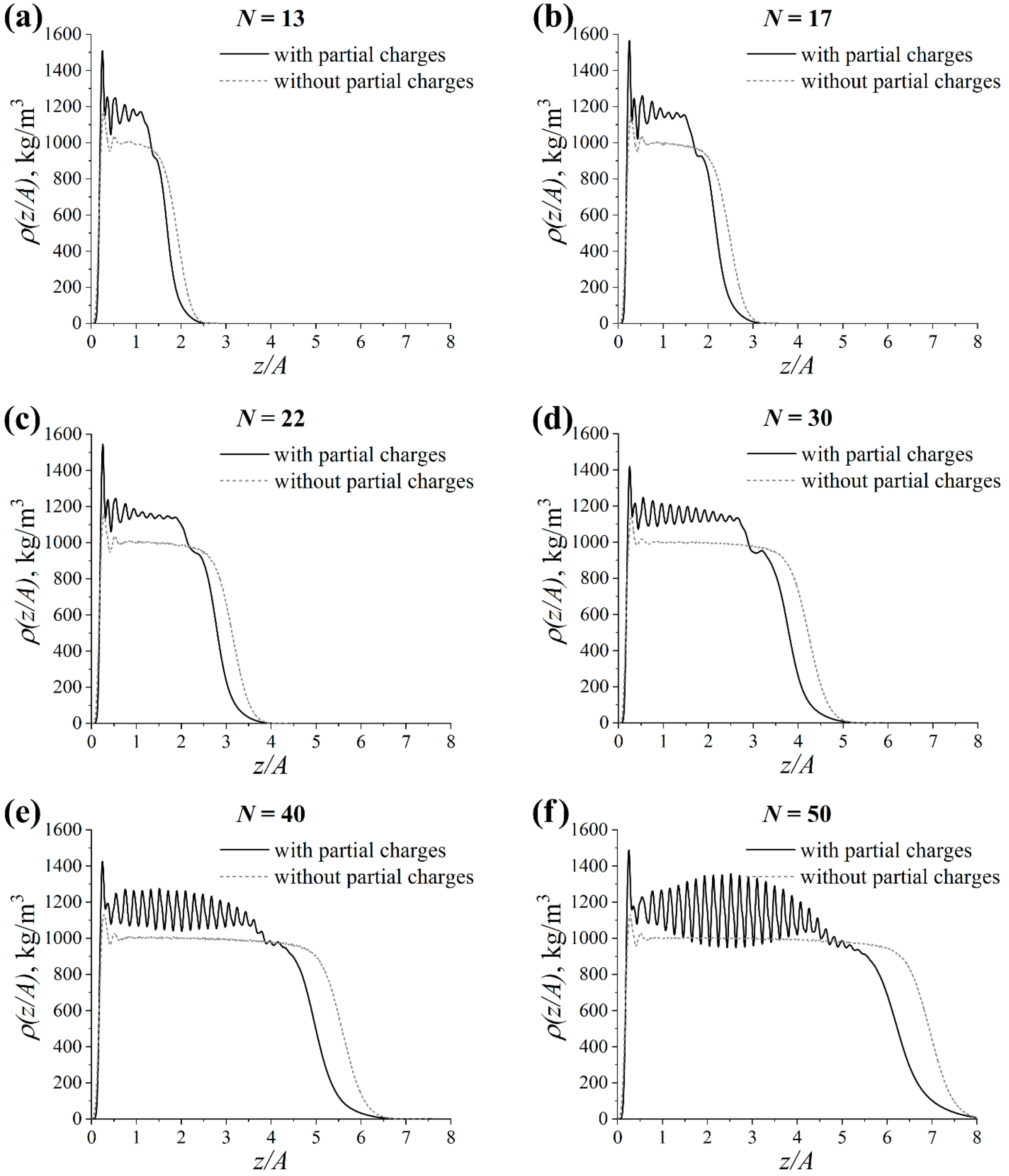

Figure S3. Normal density profiles of the grafted chains *ρ(z/A)* related to the CNC surface at different graft's length *N* in the systems with and without partial charges. *A* is the Kuhn segment length for the lactide chains.

4. Order parameter for the grafts' monomers

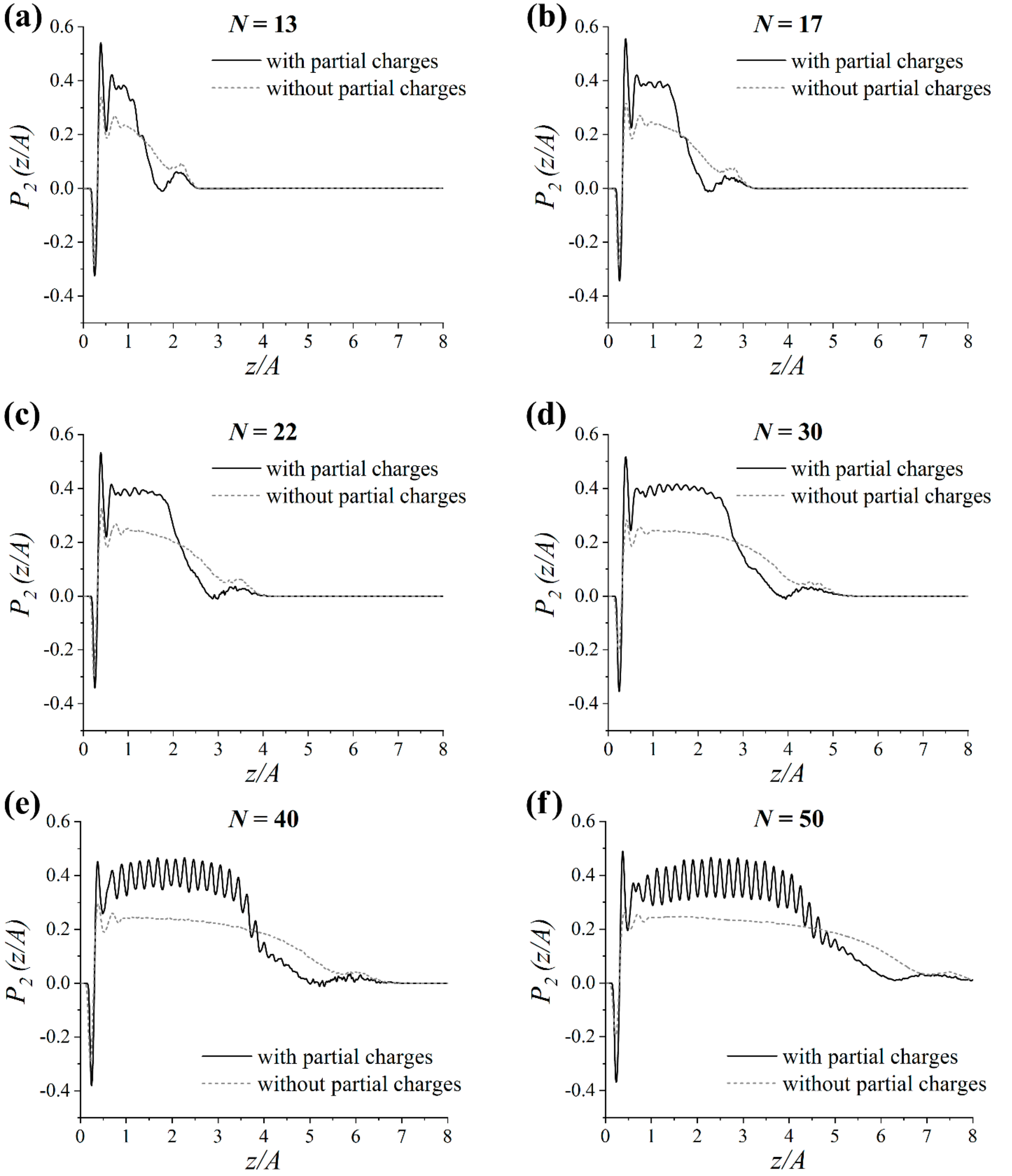

Figure S4. Order parameter $P_2(z/A)$ for the grafts' monomers as a function of their normalized distance from the filler surface z/A at different chain length N for the systems with and without partial charges. A is the Kuhn segment length for the lactide chains.

5. Lateral density profiles for the grafted chains stretched from the surface

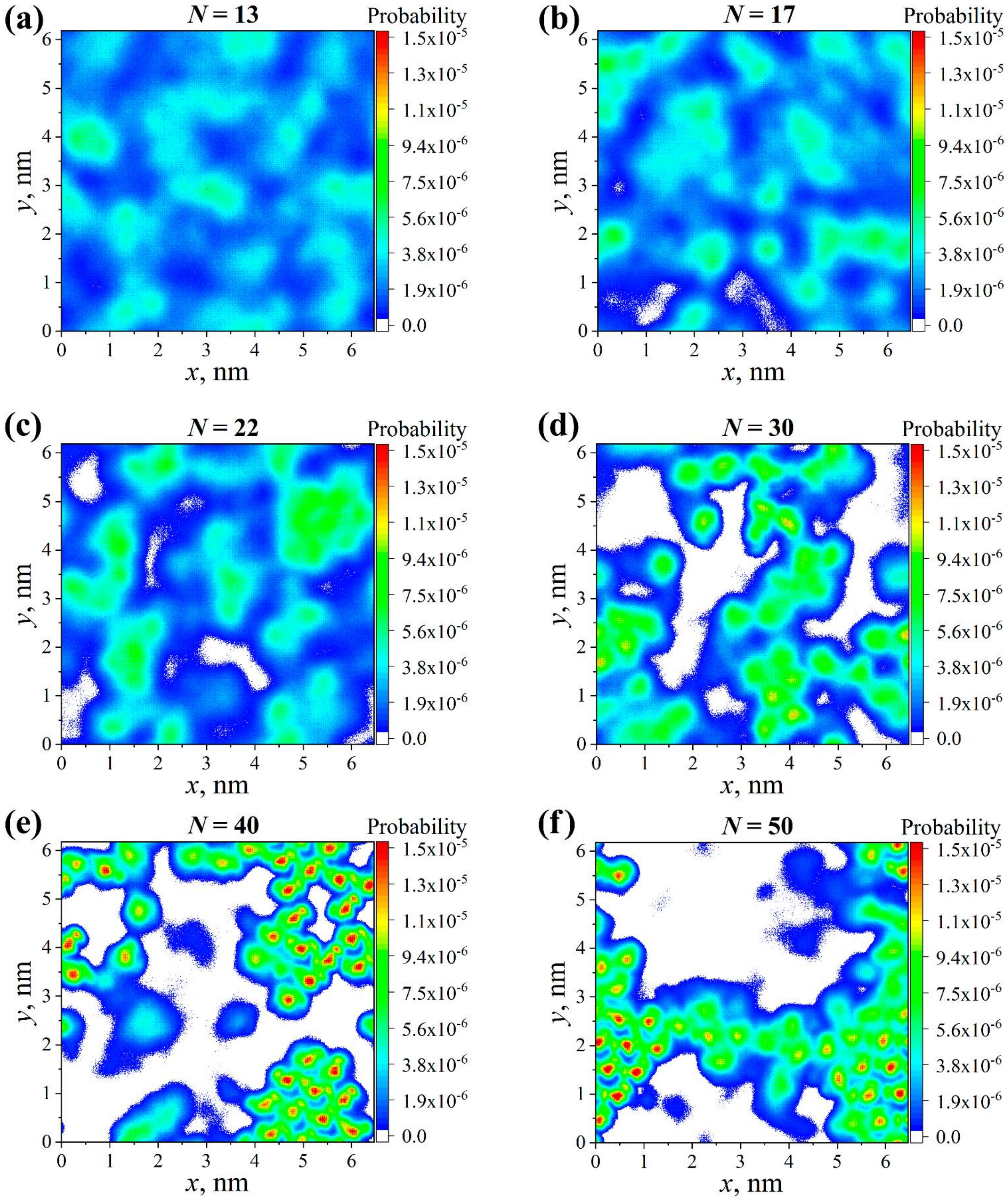

Figure S5. Lateral density profiles of the grafted chains stretched from the filler surface at different chain length *N* for the systems with partial charges.

6. Comparison of the systems with different sizes simulated in the GAFF and PLAFF force fields

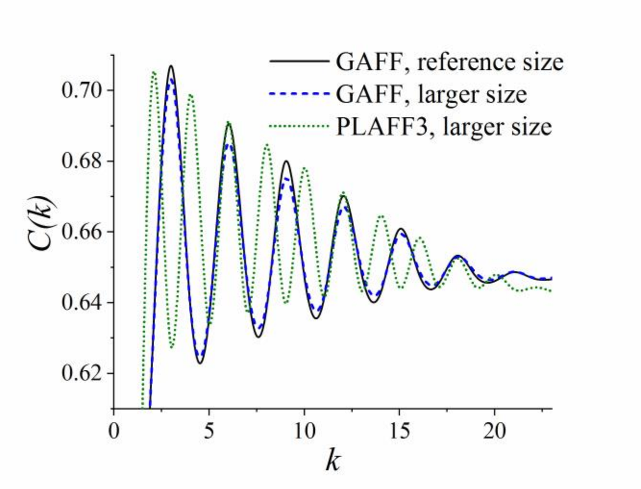

Figure S6. Autocorrelation function $C(k)$ for the grafts with the chain length $N = 50$ in the systems with reference sizes (used in the present paper) and larger sizes simulated in the GAFF and PLAFF force fields.
